# A History of the Malignant Intermittent Bilious Fever, Which Prevailed in and about Indianapolis, the Capital of the State of Indiana, in the Summer and Autumn of 1821

**Published:** 1828-12

**Authors:** Samuel Grant Mitchell

**Affiliations:** Indianapolis


WESTERN JOURNAL

OF THE

MEDICAL & PHYSICAL SCIENCES.

DECEMBER, 1828.

### ESSAYS AND CASES.

Art. I.—*A History of the Malignant Intermittent Bilious Fever, which prevailed in and about Indianapolis, the capital of the state of Indiana, in the summer and autumn of* 1821. By Dr. Samuel Grant Mitchell.

Climate, soil, modes of living, and various other common causes, exert so great an influence on febrile diseases, that I shall state what I have observed at Indianapolis on these subjects, as an introduction to the history which I am about to give.

The new capital of the new state of Indiana, is built, or rather is building, on the east bank of the west fork of White River, a principal branch of the Wabash, immediately below the mouth of Fall creek, in lat. 39° 45′, or nearly that of Philadelphia. The river side of the town plat is about half a mile from the water. It is on an extensive alluvial plain, elevated 12 or 15 feet above high water mark. This plain, previous to the year 1820, was heavily wooded with white and blue ash, walnut, hickory, cherry tree, sugar tree, linden tree, elm, mulberry, and the other trees of the fertile lands of the West; with a thick undergrowth of pawpaw, spicewood and prickly ash. It affords an abundance of pure and pleasant limestone water. The adjacent country abounding in beech timber, is level, and from the argillaceous nature of the soil, its surface is more wet than that of the bottom lands, the water of the spring rains being in many places accumulated into morasses and ponds. The town plat is dry, but adjoining to it there are two bayous from the river, which, however, might be easily drained. In digging into the plain, after passing through 4 or 5 feet of soil and loam, we come to gravel, which continues for 15 or 20 feet, after which we meet with nothing but white sand, as far as perforations have yet been made.

In the spring of the year 1821, the heavy growth of timber which covered the site of the projected town, was cut down, and most of it suffered to lie where it fell. This was done to facilitate the surveying of the lots. They were offered for sale, and immediately purchased either by speculators or actual settlers. Persons of all kinds forthwith crowded to the spot, and the number in a short time amounted to nearly six hundred; who were, of course, in general, strangers to each other, and to the climate. They were of necessity, huddled together into tents, camps, shanties, and leaky cabins. Vegetable food was scarce, and the poorer classes especially, lived chiefly on fish, venison, and other wild meats, which were eaten with very little salt, from the scarcity of that article.

I arrived in the month of July, 1821, which together with the other summer months, was unusually warm and wet. In the forenoons we very commonly had copious showers, and in the afternoons fair weather, with intense heat. In the miserable habitations of the newly arrived inhabitants, so great was the humidity, that every article of furniture and dress was covered with mould and mildew. This excess of heat and moisture gave extraordinary energy to vegetation, and at the same time contributed to a rapid decay of those plants, both woody and herbaceous, which had been either trampled or cut down. In many parts of the new town, the vegetable putrefaction, was, indeed, so rapid as to give rise to exhalations offensive to the smell. Insects of all kinds were exceedingly numerous and annoying.

On the 20th of July, I observed near the bank of the river, a number of dead cats and dogs. The inhabitants said they had died from eating fish; but I began to look out for difficult times. I repeated to my friends the couplet from Pope's Homer—

"On dogs and mules the infection first began,
And last the vengeful arrows fixed on man;"

which turned out to be prophetic; for on the 25th of the same month (a few scattering cases having previously occurred) an epidemic fever suddenly broke out. It began on the side next the river, and spread eastwardly, till like the "pestilence that walked in darkness" in ancient times, it involved our whole population. No disease could have been more general. It is said that there were only two persons in the whole town but what took medicine; and no less than seventy-two, or about an eighth part of the whole population died. The physicians suffered with the rest. My partner Dr. Dunlap and myself, both experinced violent attacks, and from premature exposure suffered several relapses.

In the month of September the fever was most malignant and fatal; early in October the nights grew cool; and some cold rains occurred during the first week, when the number of new cases rapidly diminished; and, after the eighth of that month, ceased altogether; the epidemic having, indeed, exhausted nearly all the subjects which the young town could furnish.

The disease assumed, even in its worst attacks, an intermittent type. In a great number of cases it was mild and manageable; but others presented all the characteristics of a malignant congestive fever. In its most terrible form, it was the *algid* intermittent of Alibert. Many of the most dangerous cases, however, commenced as simple intermittents.

At the very beginning of the bad cases, there was great diminution in the size of the external parts of the body; and the countenance of the patient was altered and looked more acute. The tongue varied with the stages of the paroxysm, being white, red, yellowish, and dark brown or black. The last looked like the stain of grapes, while the redness of the organ was similar to that occasioned by poke berries; and of all the appearances, gave the most unfavorable indication.

The skin in many cases was yellow; sometimes dry, but oftener cold and clammy with sweat. The patient was often soporous, and I observed that this symptom was extremely portentous when it followed the chill. This, in the worst cases returned daily, and sometimes twice a day, constituting double tertians, quotidians, and double quotidians. I saw a patient late in the evening, and said, you have a severe chill: 'yes—said he—I have a double *twister;* this is the second chill I have had since morning attended with an icy coldness; my first shake lasted four hours.' He was destitute of a pulse; but by the assiduous administration of remedies, he recovered. In some cases the patient was cold for 12 or 15 hours. In the first paroxysm, the coldness sometimes extended to the knees: in the second, to the whole body; in the third, would prove fatal. In cases of unfavourable omen, there would be a clammy state of the surface, and its insensibility would be so great, that the strongest irritants could not excite it. The arteries of the wrists would feel like a small, round, switch. They were in fact, almost empty; still this was called a corded pulse, and made the occasion for bloodletting, under the operation of which, the patient sometimes expired! The thirst and anxiety of the patient were often very great. In some instances there was a profuse perspiration, with icy coldness of the surface; as I had seen it in the pneumonia typhodes, or cold plague of 1812. This condition was highly dangerous, as Alibert had already observed. "The danger of the fever," says he, "actually increases as the sweats become more copious and general; the skin appears to be in a state of complete atony; all the pores being open, give vent to the sweat, which is thick, viscous, and cold, and so copious sometimes, as even to pass through the bed." Desperate as these cases were, our remedies often proved successful beyond the most sanguine expectations.

In the malignant cases, vomiting almost uniformly occurred, and a dark, bilious matter was thrown up. In the month of August, I found nearly every tenant of a cabin, puking at the same time. They were thirteen in number.

Such were the leading symptoms of this fever; but in different cases it concentrated itself upon various organs of the body, constituting besides the algid, soporose, and diaphoretic states of Alibert, his dysenteric, rheumatic, nephritic, and cephalalgic forms of fever. In several instances I saw it produce epileptic convulsions.

A more extended enumeration of symptoms is unnecessary, as what I have related must disclose the nature of this disease, and will therefore proceed to speak of its treatment.

In this fever the danger appeared and disappeared with the paroxysms; but in the most desperate cases there were no perfect intermissions. In the paroxysm, both the cold and the hot stages required separate and specific attentiou. In the first, I had recourse to every practicable means of excitation, both external and internal. The former were additional bed clothes, sinapisms, hot bricks, and stones, and bags of sand, salt, or ashes; but I preferred to wrap the limbs and body of my patient in flannels dipped in boiling brandy or whiskey, after having well rubbed the surface. I found heat and moisture combined much better than heat alone. As internal stimulants, we gave brandy and laudanum, in large quantities. During this stage, which sometimes continued for ten or twelve hours, I often gave a quart of fourth proof brandy with an ounce of strong laudanum, and thereby saved the patient. In the hot stage, although the skin might be warmer, and the pulse stronger, the circulation was still sluggish, even at the commencement of the attack. In this stage of the paroxysm, I constantly administered mercurial purges, in great quantities, for the bowels were costive. I frequently gave a drachm of calomel, at once, and repeated the medicine, in half drachm doses, every two hours, until large bilious discharges were produced. As auxiliaries, in the fulfilment of this indication, I never resorted to senna, castor oil, salts and other medicines of this kind; regarding them as incapable of accomplishing that which calomel could not effect. In favor of the liberal and long continued use of this medicine, I was accustomed to reason with my patients, and when I could not persuade them to agree to its further employment,

I resorted to disguises, and persevered in my plan, without their knowledge or consent. Thus I would rub half an ounce of the medicine with half a drachm of bark, and exhibit it in proper doses, as a purging powder. In this manner, I have given pounds of calomel, since I came to Indiana; and I here solemnly declare, that I have never lost one patient whose death I could ascribe to that medicine; while hundreds have been cured by myself and friends by its liberal use. True, it frequently occasioned a sore mouth, which sometimes proved dangerous, or protracted, but its beneficial operation was more than a compensation for these sinister effects. The occurrence of a salivation was not always the sign of a removal of the disease; and it seemed to be necessary to the safety of the patient, that the bilious evacuations should be kept up. To accomplish this when a sore mouth admonished me to discontinue the calomel, or I feared a violent ptyalism, I was in the habit of resorting to a powder composed of equal parts of aloes, gamboge, and rhubarb, which answered fully as a substitute for the disguised calomel. Sometimes, however, I merely added rhubarb to the bark.

For the vomiting and gastric heat, my friend Dr. Dunlap, recommended to me a medicine which he had found to succeed, perfectly, after the carbonate of potash, magnesia, and the aromatics had entirely failed; which, in fact, they generally did. It was *opium*, which produced effects sufficient to justify the epithet, 'divine remedy' applied to it by the ancients. We frequently gave from one to five grains of this medicine. It stopped the vomiting without in the smallest degree retarding the cathartic operation of the calomel.

While the calomel was still operating, I resorted to the cinchona, and found the two medicines in no degree incompatible with each other. I generally gave a drachm of good Lima bark every two hours, mixed in a glass of brandy. About one hour before the return of the icy coldness, I gave from one to three teaspoonsful of strong laudanum. It may be said, once for all, that in the intermission, Bark, brandy, and laudanum, in large doses, constituted our anchor of hope. I find in the books no examples of the large and early exhibition which we made of these powerful remedies, which I have enumerated, but their use undoubtedly saved hundreds of lives.

Few cases of this fever either required or admitted of bloodletting; and, as I have already said, there were cases in which it proved fatal. Among those which constituted exceptions to the prohibition, I may rank my own. I was bled copiously, by Dr. Dunlap, three times in 24 hours. I had a great difficulty of breathing, with a slow, hard, and irregular pulse, which was relieved by the evacuation; and the Bark, brandy, and laudanum, finished the cure. As a means of preventing relapses, I found the daily use of an infusion of wormwood, colomba or chamomile, to possess very considerable efficacy.

I wish in this place to enter my protest against the use of emetics and nauseants, in all congestive bilious fevers like that which I have described. If these fevers have an emetic stage, I have not been able to discover it. The experience of others in other places may, however, have been different; for, as I stated in the beginning, differences of climate, modes of living, &c. give rise to great diversities in the character of our fevers. In this way only can I reconcile with my own experience, that of a distinguished professor, who, in treating malignant fevers, relies exclusively on mercurial cathartics, and in the intermission does not resort to tonics. Such a practice has not succeeded with any physician of this place.

In concluding this history of a fatal epidemic, I consider it but an act of justice to the physicians of Indianapolis to say, that during its prevalence, their intercourse with each other afforded no support to the adage that 'doctors will differ.' At an early period they were led to form nearly the same opinions on its treatment, and cherishing the same zeal for the afflicted, they were united in their efforts, and contributed all in their power to sustain and encourage each other in the trying scenes through which they had to pass. These were greater, than those who have never witnessed an epidemic in the depths of a remote wilderness, can easily imagine; and could not have been successfully encountered without union and reciprocal aid.

From the year 1821, down to the present autumn, the fevers of Indianapolis have been gradually abating in their violence; and in the number of persons attacked; thus confirming an opinion that I have often expressed, that the capital of the state of Indiana is not an unhealthy town.

Our diseases are gradually becoming more inflammatory. During the present autumn we have had an epidemic bilious fever, but although our population amounts to 1200, it has proved fatal but to four grown persons, and four or five children. Our principal remedies have been the lancet and mercurial purges, the bowels being generally costive. The Bark in some instances, evidently did harm. When, however, in protracted cases, the disease assumed an intermitting type, it was speedily cured by brandy and the sulphate of quina.

Persons emigrating to Indiana, especially those who settle near the water courses, are liable the first year, to autumnal diseases; but they are generally of a mild kind, most commonly the ague and fever.

*Indianapolis, Nov.* 11, 1828.

